# Estimating Compressive Strength of Concrete Using Neural Electromagnetic Field Optimization

**DOI:** 10.3390/ma16114200

**Published:** 2023-06-05

**Authors:** Mohammad Reza Akbarzadeh, Hossein Ghafourian, Arsalan Anvari, Ramin Pourhanasa, Moncef L. Nehdi

**Affiliations:** 1 Department of Civil Engineering, Sharif University of Technology, Tehran 1136511155, Iran; 2 Department of Civil and Environmental Engineering, University of Massachusetts Amherst, Amherst, MA 01003, USA; 3 Department of Construction Engineering and Management, Faculty of Civil Engineering, Science and Research Branch, Islamic Azad University, Tehran 1477893855, Iran; 4 Department of Civil Engineering, College of Engineering, Shahrekord University, Shahrekord 64165478, Iran; 5 Department of Civil Engineering, McMaster University, Hamilton, ON L8S 4L7, Canada

**Keywords:** civil engineering, concrete compressive strength, metaheuristic strategies, electromagnetic field optimization

## Abstract

Concrete compressive strength (CCS) is among the most important mechanical characteristics of this widely used material. This study develops a novel integrative method for efficient prediction of CCS. The suggested method is an artificial neural network (ANN) favorably tuned by electromagnetic field optimization (EFO). The EFO simulates a physics-based strategy, which in this work is employed to find the best contribution of the concrete parameters (i.e., cement (*C*), blast furnace slag ($S_{BF}$), fly ash (${FA}_{1}$), water (*W*), superplasticizer (*SP*), coarse aggregate ($A_{C}$), fine aggregate (${FA}_{2}$), and the age of testing ($A_{T}$)) to the CCS. The same effort is carried out by three benchmark optimizers, namely the water cycle algorithm (WCA), sine cosine algorithm (SCA), and cuttlefish optimization algorithm (CFOA) to be compared with the EFO. The results show that hybridizing the ANN using the mentioned algorithms led to reliable approaches for predicting the CCS. However, comparative analysis indicates that there are appreciable distinctions between the prediction capacity of the ANNs created by the EFO and WCA vs. the SCA and CFOA. For example, the mean absolute error calculated for the testing phase of the ANN-WCA, ANN-SCA, ANN-CFOA, and ANN-EFO was 5.8363, 7.8248, 7.6538, and 5.6236, respectively. Moreover, the EFO was considerably faster than the other strategies. In short, the ANN-EFO is a highly efficient hybrid model, and can be recommended for the early prediction of the CCS. A user-friendly explainable and explicit predictive formula is also derived for the convenient estimation of the CCS.

## 1. Introduction

Analyzing buildings from different perspectives is of great importance for safe design [1,2,3]. Concrete is among the most widely used materials for construction purposes [4,5,6]. It is used for building various structural elements, from foundations to columns and beams [7,8]. Hereupon, engineers have conducted studies to analyze the behavior of concrete in terms of various parameters, such as ion penetration resistance [9], seismic behavior [10], load-bearing capacity [11], slump and workability [12], etc.

High-performance concrete (HPC) is a strong and durable type of concrete that has gained significant popularity for many construction projects all over the world [13,14,15]. In concrete, compressive strength is the main design parameter [16,17]. Therefore, evaluating this parameter is of great importance to structural and geotechnical engineers. It is an essential yet difficult task [18,19]. With that said, the non-linear abilities of machine learning methods make them a suitable solution to such issues [20,21,22]. They can effectively explore the influence of various key factors on concrete compressive strength (CCS).

Data-mining techniques, such as gene expression programming [23], support vector machine (SVM) [24], and artificial neural network (ANN) [25], have broadly served as the approximators of CCS. Dao et al. [26] examined the efficiency of ANN and a Gaussian process regression (GPR) developed using various kernels for predicting the strength of HPC. In this way, they also used Monte Carlo simulation to investigate the sensitivity of the models. Evaluation of the accuracy of the implemented models showed that the GPR with Matern32 function yields the best prediction. Shahmansouri et al. [27] modeled the CCS of pozzolanic geopolymer concrete based on ground granulated blast-furnace slag. High agreement between the products of the ANN and expected CCSs confirmed the reliability of the proposed technique. Jalal et al. [28] proved the applicability of an adaptive neuro-fuzzy inference system (ANFIS), which is a powerful approximator for estimating the strength of rubberized concrete measured at four different ages (3, 7, 28, and 42 days). Dao et al. [29] studied the suitability of the ANN for CCS prediction in foamed concrete. Based on 97.2% testing correlation obtained for the optimized structure, they introduced the ANN as an efficient predictor of this parameter. Additionally, the executed sensitivity analysis revealed the largest impact for the dry density factor. Simsek et al. [30] incorporated a decision support method with extreme gradient boosting (XGB) to create an efficient predictor for the strength of HPC. Regarding the root mean square errors (RMSEs) of 4.36, 7.15, 10.47, and 6.71 calculated for the XGB, MLR, R-MLR, and ANN, the XGB was the superior model. Further studies with a focus on utilizing machine learning models in this field can be found in [31,32,33,34].

In more recent studies, scholars have realized that metaheuristic algorithms can be used for design aims [35,36,37]. Ulusoy et al. [38], for example, employed metaheuristic algorithms such as bat algorithm and harmony search for the optimal design of reinforced concrete beams. Moreover, metaheuristic strategies have excellent search capacities for attaining optimal solutions to a problem. Converting the prediction tasks (using data-mining techniques) to an optimization problem, these algorithms are capable of finding the optimal situation for the processor [39,40]. Hoang et al. [41] optimized a least squares support vector regression (LSSVR) using a differential flower pollination algorithm for simulating concrete bond strength. The RMSE = 2.39 and R^2^ = 0.84 indicated a high accuracy for the proposed model and its superiority over conventional tools such as ANN. Mai et al. [42] were able to enhance the accuracy of the radial basis function ANN using a firefly algorithm (FFA) for predicting the axial compression capacity of concrete-filled steel columns. Two other metaheuristic techniques, namely genetic algorithm (GA) and differential evolution (DE), which were relatively weaker than FFA, were also used. Ma et al. [43] evaluated the efficiency of four metaheuristic optimizers, namely a salp swarm algorithm (SSA), grasshopper optimization algorithm (GOA), artificial bee colony (ABC), and shuffled frog leaping algorithm (SFLA), to tune the ANN for predicting the CCS. Considering the time and accuracy measures, the SSA and GOA were introduced as the strongest algorithms. In this regard, a notable distinction was observed for the correlation between these two algorithms (around 0.97%) and ABC (70.60%) and SFLA (88.90%). The predictive formula of the SSA and GOA was lastly derived. Notably, ABC emerged as a time-consuming technique. The use of the whale optimization algorithm (WOA) for the same purpose (i.e., neural network tuning for the CCS modeling) was recommended by Bui et al. [44]. The dragonfly algorithm and ant colony optimization were two other optimizers that presented a weaker performance relative to the WOA. A hybrid of the random forest and beetle antennae search (BAS) algorithm was proposed by Zhang et al. [45] for simulating the uniaxial compressive strength of oil palm shell concrete. Due to the high correlation (>95%) obtained for the testing process, the suggested model is a reliable and effective approach to the aforementioned simulation.

As we have argued, diverse efforts concerning the accuracy improvement of conventional approaches illustrate the importance of this task in civil engineering. On the other hand, benefiting from optimization strategies (e.g., grey wolf optimizer [46], symbiotic organism searches [47], teaching learning-based optimization [48], etc.) has been regarded an effective idea for this purpose. Hence, this study is concerned with the evaluation of an innovative hybrid method for predicting the CCS of HPC. To this end, electromagnetic field optimization (EFO) is combined with an ANN model to offer an optimal predictor. Moreover, the EFO is validated through comparing the results with three other optimizers, namely the water cycle algorithm (WCA), sine cosine algorithm (SCA), and cuttlefish optimization algorithm (CFOA).

## 2. Materials and Methods

### 2.1. Data Provision

**Figure A1:**
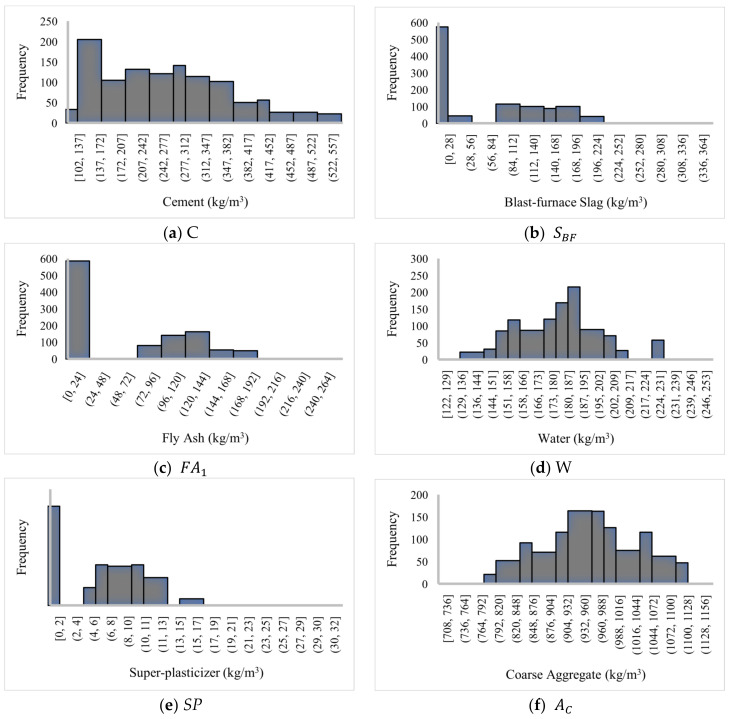

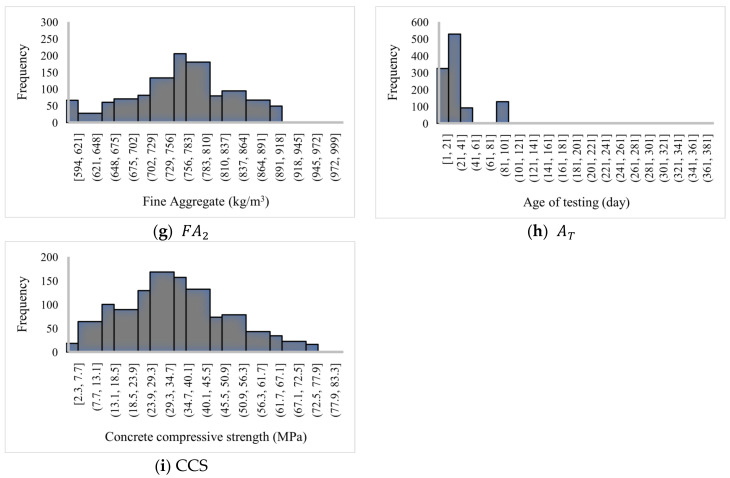
Histogram of the dataset.

The used dataset consists of compressive strength records of 1133 HPC samples [49]. It was originally created by Yeh [50], and has also been used in earlier studies [51,52]. Observing these data, the CCS is affected by the present amounts of seven ingredients: cement (*C*), blast furnace slag ($S_{BF}$), fly ash (${FA}_{1}$), water (*W*), superplasticizer (*SP*), coarse aggregate ($A_{C}$), fine aggregate (${FA}_{2}$), and the age of testing ($A_{T}$). Figure A1 shows a histogram of the data.

Table A1 illustrates the dataset statistically. Regarding $A_{T}$ values, the specimens are 1, 3, 7, 14, 28, 56, 90, 91, 100, 120, 180, 270, 360, and 365 day(s) old. The weakest and strongest specimens are distinguished by the minimum and maximum CCSs, 2.33 and 82.60 MPa, respectively. The average CCS of the whole dataset is 35.84 MPa. Likewise, the mean value for *C*, $S_{BF}$, ${FA}_{1}$, *W*, *SP*, $A_{C}$, ${FA}_{2}$, and $A_{T}$ is 276.50 kg/m^3^, 74.27 kg/m^3^, 62.81 kg/m^3^, 182.98 kg/m^3^, 6.42 kg/m^3^, 964.83 kg/m^3^, 770.49 kg/m^3^, and 44.06 days, respectively.

Of these 1133 samples, first, 906 samples are randomly selected to feed the networks. They use these data for learning the relationship between the target parameter (i.e., the CCS) and the inputs (i.e., *C*, $S_{BF}$, ${FA}_{1}$, *W*, *SP*, $A_{C}$, ${FA}_{2}$, and $A_{T}$). More clearly for the metaheuristic hybrids used in this study, the WCA, SCA, CFOA, and EFO assign a multiplying weight to each input factor, and a bias term to each neuron of the ANN. The same process is carried out for the subsequent neurons to create a non-linear model. The acquired pattern is then exposed to the rest of the data, which comprises 227 samples, to predict the CCS for unfamiliar specimens. In this way, the versatility of the models is assessed.

### 2.2. EFO Algorithm

The EFO is considered a physics-based technique because the developers, Abedinpourshotorban et al. [53], used electromagnetics rules for designing it. The population of this algorithm is cooperative, and aims to improve the position for the betterment of the solution. Attraction–repulsion forces affect individuals called electromagnet particles (EMPs). Famously, opposite, and similar polarities cause attraction and repulsion forces, respectively. For EMPs, there is more power in the attraction forces (5–10% higher than the repulsion forces). In the EFO, a golden value is sought for the ration between these forces. During the implementation, the EMPs in the neutral field maintain distance from those of the negative field (i.e., the poorly fitted ones) because of the repulsion interaction, and conversely, they approach those of the positive field (i.e., nicely fitted ones) because of the attraction interaction.

First, a population is initialized, evaluated, and sorted according to the individuals’ fitness. They are classified into three groups, namely the positive field (best individuals), negative field (weak individuals), and neutral field (the rest). In the neutral field, there are small negative polarities close to zero, where

(1) $$N_{+ve}=P\_field.N\_emp,$$

(2) $$N_{-ve}=N\_field.N\_emp,$$

(3) $$N_{neutral}=N\_emp-(N_{+ve}+N_{-ve}),$$

where $N_{+ve}$, $N_{-ve}$, and $N_{neutral}$ symbolize the population of the negative, positive, and neutral groups. Likewise, N_emp stands for the whole population. Additionally, P_field and N_field are the EFO parameters.

In the subsequent step, a new individual is created, and after comparing its fitness with existing ones, it finds the appropriate place, and the polarity is accordingly determined. Remarkably, the weakest individual is removed from the population. The generation of the new individual can be modeled as follows:

(4) $$D_{m}^{N_{m}K_{m}}={EMP}_{m}^{r_{negative}}-{EMP}_{m}^{r_{neutral}},$$

(5) $$D_{m}^{P_{m}K_{m}}=EMP_{m}^{r_{positive}}-EMP_{m}^{r_{neutral}},$$

(6) $$EMP_{m}^{new}=EMP_{m}^{r_{neutral}}+\lambda .rand.D_{m}^{P_{m}K_{m}}-Force.D_{m}^{N_{m}K_{m}}\;,$$

where λ is the golden ration, rand is a random value in ♥0, 1♥, m = 1, 2, 3, …, and N_var, $r_{positive}\in \;\left\{1,\;2,\;\ldots ,\;j\right\}$, $r_{neutral}\in \;\left\{1+j,\;2+j,\;\ldots ,\;z\right\}$, and $r_{negative}\in \;\left\{1+z,\;2+z,\;\ldots ,\;N\right\}$ give random integers where $z=N_{+ve}+N_{neutral}$ and $j=N_{+ve}$ [54]. Further sources for learning about the EFO mechanism are [55,56,57].

### 2.3. Benchmarks Optimizers

As previously mentioned, the EFO is validated by comparing its performance with three other algorithms, namely WCA, SCA, and CFOA. These techniques were designed by Eskandar et al. [58], Mirjalili [59], and Eesa et al. [60], respectively. As a generic measure in population-based optimizers, the algorithms go through certain steps (e.g., initializing the population, fitness evaluation, updating the situation, and monitoring stopping criteria) to perform the optimization task. 

For example, the water cycle (and the movements of rivers and streams toward a sea) is the inspiration for the WCA algorithm. After it has rained, the raindrops are evaluated and based on their goodness, and they form streams, rivers, and one sea so that the most accurate solution is held in the sea. Other components update their positions to improve themselves and replace the sea. This is while the SCA is based on mathematical rules including sine and cosine functions. Utilizing some random values, the position of the population is improved to obtain a more promising solution. Unlike the WCA and SCA, the CFOA is a bio-inspired method that simulates the camouflage action of the cuttlefish. Over two stages, namely reflection and visibility, which mimic the light reflection and pattern matching procedures, respectively, this animal is able to enact an optimal change in its color. Detailed descriptions of the WCA, SCA, and CFOA can be found in references [61,62,63,64,65,66], respectively.

### 2.4. Quality Measures

In comparable earlier studies, accuracy is the most important determinant factor in the efficiency evaluation of the models. Therefore, proper measures should be deployed for reporting accuracy. In this paper, the errors of both learning and prediction are measured using two well-known criteria, namely the root mean square error (RMSE) and mean absolute error (MAE), where the error indicates the differences between the laboratory and estimated CCSs (i.e., Error = $CCS_{i_{\mathrm{Laboratory}}}-CCS_{i_{\mathrm{Estimate}}}$). As the names connote, they give a rooted value of the squared average of the errors, and the average of the absolute errors, respectively. Calculations of the RMSE and MAE are shown in Equations (7) and (8).

(7) $$RMSE=\sqrt{\frac{1}{Z}{\sum_{i=1}^{Z}\left[(CCS_{i_{Laboratory}}-CCS_{i_{Estimate}})\right]}^{2}}$$

(8) $$MAE=\frac{1}{Z}\sum_{I=1}^{Z}\left|CCS_{i_{Laboratory}}-CCS_{i_{Estimate}}\right|$$

where *Z* stands for the size (i.e., the number) of the assessed data.

The Pearson correlation coefficient (PCC) is a correlation evaluative index that is used herein to report the agreement between the $CCS_{i_{\mathrm{Laboratory}}}$. and $CCS_{i_{\mathrm{Estimate}}}$ for all *Z* samples. The PCC is formulated as follows:

(9) $$PCC=\frac{\overset{Z}{\underset{i=1}{-}}\left(CCS_{i_{Estimate}}-{\bar{CCS}}_{Estimate})(CCS_{i_{Laboratory}}-{\bar{CCS}}_{Laboratory}\right)}{\sqrt{\overset{Z}{\underset{i=1}{-}}{\left(CCS_{i_{Estimate}}-{\bar{CCS}}_{Estimate}\right)}^{2}}\sqrt{\overset{Z}{\underset{i=1}{-}}{\left(CCS_{i_{Laboratory}}-{\bar{CCS}}_{Laboratory}\right)}^{2}}}$$

## 3. Results and Discussion

### 3.1. Optimization and Training Assessment

As was mentioned earlier, this study offers various metaheuristic trained ANNs for predicting CCS. The training process is detailed in this section. First, a general structure of the ANN with adjustable parameters was coded and given to the WCA, SCA, CFOA, and EFO as the problem function. In each iteration of implementing the ANN-WCA, ANN-SCA, ANN-CFOA, and ANN-EFO, the algorithm aims to minimize the RMSE of training. In other words, this criterion is deemed the objective function. This is how the optimal ANN is achieved by each optimizer [67,68].

**Figure 1:**
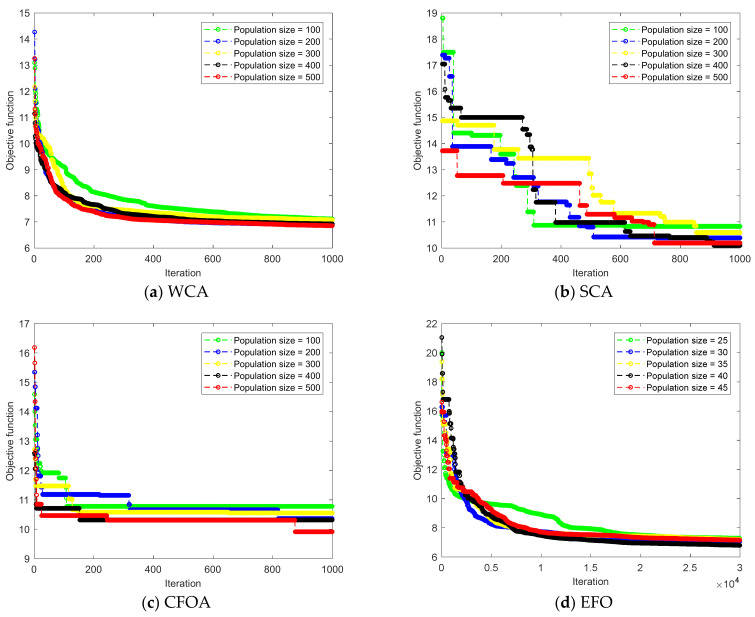
Optimizing the ANN using different metaheuristic configurations.

The number of the iterations (*N_Iter_*) and population size (*S_Pop_*) are two important parameters of all metaheuristic algorithms that need to be selected carefully. Both parameters can be completely different for two algorithms, due to the specific behaviors and strategies executed by them. For the EFO, five *S_Pop_*s (25, 30, 35, 40, and 45) were tested, where the *N_Iter_* equals 30,000, while the WCA, SCA, and CFOA were tested using *S_Pop_*s of 100, 200, 300, 400, and 500, with *N_Iter_* = 1000 [69,70]. In this way, the sensitivity of the algorithms to *S_Pop_* can be monitored, and the best network configuration can be selected. Figure 1 shows the convergence curves obtained after this process. According to this figure and based on the error reduction steps, the optimization proceedings of the algorithms are different from each other. The CFOA, for example, reduced the objective function in the initial iterations more effectively, while the SCA reduced it more gently, in several steps, and the EFO and WCA reduced it by following a smooth trajectory.

**Figure 2:**
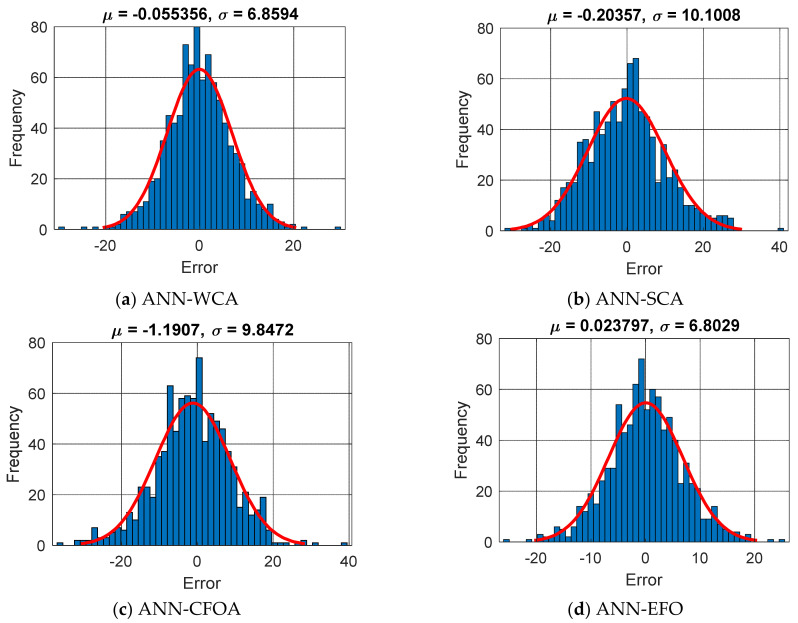
The frequency of errors in the training process (Error = $CCS_{i_{\mathrm{Laboratory}}}-CCS_{i_{\mathrm{Estimate}}}$).

This figure also shows that the best ANN training (i.e., the smallest RMSEs) was attained by the *S_Pop_* of 500, 400, 500, and 40 for the WCA, SCA, CFOA, and EFO, respectively. The obtained RMSEs of ANN-WCA, ANN-SCA, ANN-CFOA, and ANN-EFO were 6.8558, 10.0972, 9.9135, and 6.7992. The magnitude of the error was calculated for all 906 training specimens, and their frequencies are depicted in Figure 2. It is shown that all four models have suitable error histograms, meaning that compared to large errors, much larger frequencies can be observed for small errors. This reliability can be also proven by tolerable MAEs 5.2712, 7.9139, 7.6845, and 5.2653, as well as the PCCs of 0.90493, 0.79004, 0.79200, and 0.90659.

According to the above results, although all algorithms could tune the ANN properly, the search strategies of the WCA and EFO were more successful than those of the SCA and CFOA. This can be deduced from the lower errors and the higher PCC values obtained for the ANN-WCA and ANN-EFO. For the same reason, the accuracy of the EFO was slightly more promising than that of the WCA. The ANN network optimized by this algorithm is presented in the form of the below equations:

(10) $$H_{1}=\frac{2}{1\;+\;e^{-2\left(\;-\;0.3264\;\times \;C\;-\;0.2740\;\times \;S_{BF}\;-\;1.0596\;\times \;FA_{1}\;+\;0.7545\;\times \;W\;+\;0.1109\;\times \;SP\;+\;0.6187\;\times \;A_{C}\;-\;0.5830\;\times \;FA_{2}\;+\;0.7613\;\times \;A_{T}\;+\;1.7855\right)}}-1,$$

(11) $$H_{2}=\frac{2}{1+e^{-2\left(\;-\;0.2127\;\times \;C\;+\;0.5563\;\times \;S_{BF}\;+\;1.0559\;\times \;FA_{1}\;+\;0.1903\;\times \;W\;-\;0.9102\;\times \;SP\;-\;0.4461\;\times \;A_{C}\;-\;0.8011\;\times \;FA_{2}\;+\;0.1147\;\times \;A_{T}\;+\;1.1903\;\right)}}-1,$$

(12) $$H_{3}=\frac{2}{1\;+\;e^{-2\left(\;-\;0.5947\;\times \;C\;-\;0.0047\;\times \;S_{BF}\;+\;0.5382\;\times \;FA_{1}\;+\;0.6811\;\times \;W\;+\;0.4513\;\times \;SP\;-\;0.7234\;\times \;A_{C}\;+\;1.1625\;\times \;FA_{2}\;+\;0.0497\;\times \;A_{T}\;+\;0.5952\right)}}-1,$$

(13) $$H_{4}=\frac{2}{1+e^{-2\left(\;-\;0.4336\;\times \;C\;-\;0.6956\;\times \;S_{BF}\;+\;0.2728\;\times \;FA_{1}\;+\;0.5164\;\times \;W\;+\;1.2446\;\times \;SP\;+\;0.1186\;\times \;A_{C}\;-\;0.7477\;\times \;FA_{2}\;+\;0.2306\;\times \;A_{T}\;+\;0.0000\right)}}-1,$$

(14) $$H_{5}=\frac{2}{1\;+\;e^{-2\left(0.3521\;\times \;C\;+\;1.1393\;\times \;S_{BF}\;-\;0.3272\;\times \;FA_{1}\;+\;0.3139\;\times \;W\;+\;1.0856\;\times \;SP\;+\;0.4469\;\times \;A_{C}\;+\;0.2388\;\times \;FA_{2}\;-\;0.3543\;\times \;A_{T}\;+\;0.5952\right)}}-1,\;$$

(15) $$H_{6}=\frac{2}{1\;+\;e^{-2\left(\;-\;0.0202\;\times \;C\;-\;0.8401\;\times \;S_{BF}\;-\;0.8566\;\times \;FA_{1}\;+\;0.7432\;\times \;W\;+\;0.1664\;\times \;SP\;+\;0.3674\;\times \;A_{C}\;+\;0.9189\;\times \;FA_{2}\;+\;0.4347\;\times \;A_{T}\;-\;1.1903\right)}}-1,\;$$

(16) $$H_{7}=\frac{2}{1+e^{-2\left(0.1780\;\times \;C\;+\;0.4728\;\times \;S_{BF}\;+\;0.5580\;\times \;FA_{1}\;-\;1.0040\;\times \;W\;-\;0.6533\;\times \;SP\;-\;0.9710\;\times \;A_{C}\;-\;0.2827\;\times \;FA_{2}\;+\;0.4049\;\times \;A_{T}\;+\;1.7855\right)}}-1$$

(17) $$CCS=0.0547\times H_{1}+0.9061\;\times \;H_{2}\;-\;0.9388\;\times \;H_{3}\;-\;0.3034\;\times \;H_{4}-\;0.9343\;\times \;H_{5}-\;0.9365\;\times \;H_{6}-\;0.4941\;\times \;H_{7}-\;0.5837,$$

Given that $Tansig\;\left(x\right)=\frac{2}{1+e^{-2x}}-1$ is the skeleton of Equations (10)–(16), they yield the outputs of the middle layer of the ANN. These parameters are obtained by processing the input factors (i.e., *C*, $S_{BF}$, $FA_{1}$, *W*, *SP*, $A_{C}$, $FA_{2}$, and $A_{T}$), and themselves play the role of inputs for the next layer. The final calculation is carried out using Equation (17) for releasing the predicted CCS.

### 3.2. Testing Performance

**Figure 3:**
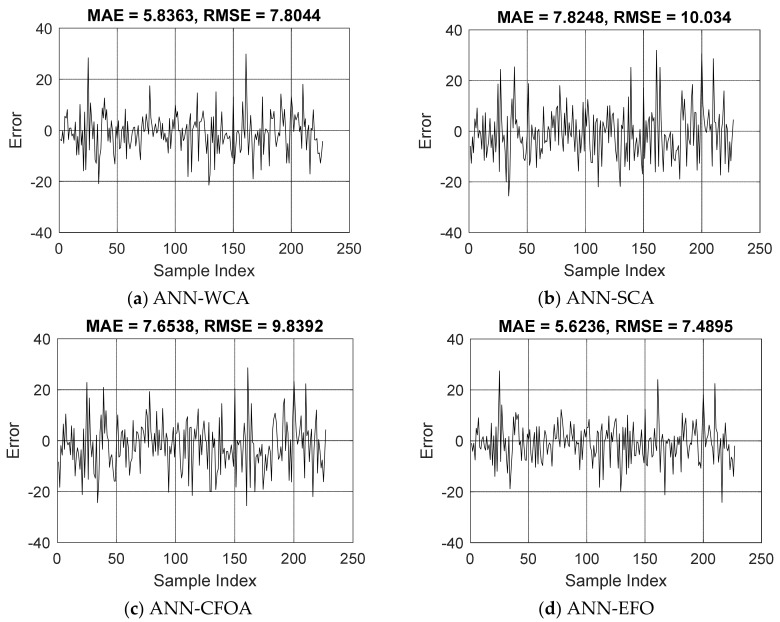
The trend of errors over the testing dataset (Error =$CCS_{i_{\mathrm{Laboratory}}}-CCS_{i_{\mathrm{Estimate}}}$ ).

As explained earlier, once the hybrids acquire a satisfying understanding from the training data, they are asked to predict the CCS for 227 stranger specimens. This work, which is referred to as the testing phase, reflects the competency of the models in dealing with new concrete mixtures. Similar to the training phase, the accuracy of prediction is examined by means of the RMSE, MAE, and PCC. Figure 3 shows the error values in this dataset. The RMSEs were 7.8044, 10.0340, 9.8392, and 7.4895, which along with the MAEs of 5.8363, 7.8248, 7.6538, and 5.6236 indicate the promising prediction potential of the used models. However, in accordance with training results, the ANNs designed by the WCA and EFO can predict the CCS with a considerably smaller error compared to those tuned with the SCA and CFOA.

**Figure 4:**
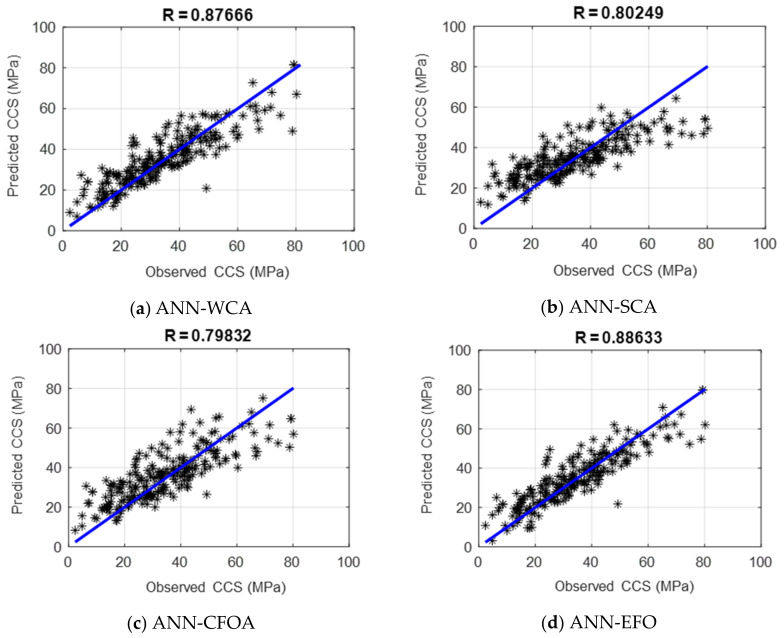
The correlation of the testing results (R = PCC ).

Moreover, the consistency of the testing results is graphically shown in Figure 4. Accordingly, the predicted CCSs are in a very good agreement with the values recorded in the laboratory. By comparison, the results of the ANN-SCA and ANN-CFOA are more scattered than the ANN-WCA and ANN-EFO. This excellence can also be demonstrated numerically in the PCCs of 0.87666, 0.80249, 0.79832, and 0.88633.

### 3.3. Discussion and More Evaluation

Civil engineering comprises a wide range of subjects from geotechnical to structural engineering, each exploring concrete from a different perspective [71,72]. Structural engineers have long considered concrete as a potential versatile material for different projects. Hence, significant attention has been paid to analyzing the behavior of concrete-based elements [73,74]. The main motivation of this research was to develop a predictive framework for analyzing the CCS of concrete. Different intelligent models were used and assessed using three accuracy indicators. Table 1 summarizes the calculated accucracy criteria. 

Evaluating the accuracy pointed out the superiority of the WCA and the proposed EFO in solving the given CCS problem. Since the ANNs tuned by these two algorithms were of higher potential than the SCA and CFOA, it can be inferred that the computational biases and weights offered by the WCA and EFO are more suitable. Therefore, these techniques enjoy excellent search abilities.

Moreover, assessing the computation times revealed that optimizing the ANN using WCA, SCA, CFOA, and EFO takes around 7520, 17443, 6430, and 314 s. In spite of the huge number of iterations considered for the EFO, it is much faster than other tested algorithms. Therefore, it may be an effective optimizer for engineering tasks in which time is a critical parameter.

Similar metaheuristic algorithms have been used in some previous studies with compatible datasets similar to the present work. These studies have proven that the tested hybrid ANNs were more accurate than conventional ANN. For instance, Hu et al. [75] were able to optimize the ANN using a multi-verse optimizer (MVO), shuffled complex evolution (SCE), and beetle antennae search (BAS). The calculated testing RMSEs were 8.3540, 7.8965, and 7.5401 for the SCE, MVO, and BAS, which are higher than the RMSEs achieved in this study by the WCA and EFO. Likewise, Moayedi et al. [76] were able to improve the accuracy of the ANN using metaheuristic models, and when comparing the accuracy, the outstanding models of the present study are more reliable than the equilibrium optimizer (RMSE = 7.8720 and MAE = 5.9869) used in the cited work. Further comparisons can be considered by referring to similar studies such as that of Li and Wu [77] (the improved sparrow algorithm), and Bui et al. [44] (the ant colony optimization and dragonfly algorithm).

From a practical point of view, there are various benefits of using the proposed models in the concrete and construction industries. These predictive models can estimate the performance of different concrete mixtures, and engineers can quickly explore a wide range of design options and select the most promising ones. Optimizing the composition of concrete materials through identifying the relationships between proportions, material components, and desired properties is another viable use; this may also be helpful in minimizing costs and environmental impacts. The EFO algorithm proposed in this work is among the quickest optimizers, and is recommended for practical estimations in cases wherein time is a critical resource. In the context of concrete and structural design, quality control, structural performance prediction, and developing decision support systems are other feasible applications of these inexpensive and time-effective metaheuristic models.

## 4. Conclusions

As the primary aim of many civil engineers, accurate simulation of CCS is of great importance for optimizing the construction process. In this work, an efficient novel predictive model was developed for this problem. Electromagnetic field optimization was synthesized with an ANN model for predicting CCS using a comprehensive and robust dataset. The sufficient learning of the CCS pattern was reflected by the MAE of 5.2653. Among the tested benchmarks, the WCA could train the ANN with superior accuracy (MAE = 5.2712). Applying the proposed model to new data revealed that the ANN-EFO can produce a reliable prediction of the CCS. The MAE in this phase was 5.6236, which is lower than those obtained for the WCA (5.8363), SCA (7.8248), and CFOA (7.6538). Apart from the accuracy, assessing the complexity of the models showed that the EFO needed a very smaller number of individuals (i.e., *S_Pop_* of 40 vs. 500, 400, and 500) for optimizing the ANN. Moreover, despite the EFO being implemented with a *N_Iter_*s 30 times that of other algorithms, it was the fastest algorithm. Therefore, the ANN-EFO may be a potent indirect method for evaluating CCS. Comparisons with some previous studies demonstrated the superiority of the models offered in this work. Additionally, recommendations were presented for practical usages of the models in the concrete and construction industries. However, the authors believe that this work can be built upon by extending the methodologies, optimizing the dataset, and performing cross-validation with real-world concrete data.

## Figures and Tables

**Table 1:** Statistics of the data.

	Training				Testing			
	ANN-WCA	ANN-SCA	ANN-CFOA	ANN-EFO	ANN-WCA	ANN-SCA	ANN-CFOA	ANN-EFO
RMSE	6.8558	10.0972	9.9135	6.7992	7.8044	10.0340	9.8392	7.4595
MAE	5.2712	7.9139	7.6845	5.2653	5.8363	7.8248	7.6538	5.6236
PCC	0.90493	0.79004	0.79200	0.90659	0.87666	0.80249	0.79832	0.88633

## Data Availability

The used data is available from the resources given in Section 2.1.

## Appendix A

**Table A1:** Statistics of the data.

Parameter	Unit	Mean	Standard Error	Standard Deviation	Sample Variance	Skewness	Minimum	Maximum
*C*	kg/m^3^	276.50	3.07	103.47	10,706.03	0.53	102.00	540.00
$S_{BF}$		74.27	2.50	84.25	7097.52	0.77	0.00	359.40
$FA_{1}$		62.81	2.13	71.58	5124.15	0.61	0.00	260.00
*W*		182.98	0.65	21.71	471.49	0.09	121.75	247.00
*SP*		6.42	0.17	5.80	33.60	0.84	0.00	32.20
$A_{C}$		964.83	2.46	82.79	6853.89	−0.17	708.00	1145.00
$FA_{2}$		770.49	2.36	79.37	6300.21	−0.19	594.00	992.60
$A_{T}$	Day	44.06	1.80	60.44	3653.15	3.47	1.00	365.00
CCS	MPa	35.84	0.48	16.10	259.23	0.42	2.33	82.60
